# Influence of duration untreated psychosis in clinical and functional outcomes at 3 months in a cohort of first episode psychosis patients

**DOI:** 10.1192/j.eurpsy.2025.2244

**Published:** 2025-08-26

**Authors:** R. Obeso Menéndez, R. Pérez Iglesias, P. Suárez Pinilla, D. Sierra Biddle, M. L. Ramírez Bonilla, P. Cordero Andrés, M. D. C. Flor Gómez, J. Vázquez Bourgon

**Affiliations:** 1 Psychiatry, Hospital Universitario Marqués de Valdecilla, Santander, Spain

## Abstract

**Introduction:**

A large number of studies have suggested that longer duration of untreated psychosis (DUP) is associated with poor clinical and functioning outcomes.

**Objectives:**

The aim of this study is to explore the association of duration of untreated psychosis and functional and clinical outcomes at short term (3 months) in first episode patients.

**Methods:**

This is a study of all patients admitted to the EI Service of University Hospital Marqués de Valdecilla in Spain, from January 2020 to July 2024, residents in the area (310,000 habitants), aged from 17 to 65, who experiencing a first episode of psychosis. We set the DUP cut-off point at 6 months to compare both groups, short-duration and long-duration psychosis. The response to treatment was assessed at 3 months with standardized scales: the PANSS scale was used to measure clinical response and the GAF scale to asses functional outcomes.

**Results:**

A total of 207 first episode patients were referred to the Early intervention Service (EIS). The mean age was 37 years-old. 54% were woman (n=111). 21% were living alone. 32% were unemployed. Forty percent (n=82) have a psychiatric family history. 63% required hospital admission and forty-nine percent were involuntary.

The mean GAF at the initial assessment was 34.8 (SD: 12.08). The mean duration of untreated psychosis (DUP) was 15 months (SEM ±2.63) and the median was 3 months (SD: 37.87). A total of 67 patients had DUP longer than 6 months.

We did not found significant differences in sex (51.4% women in the short-DUP and 58.2% women in the long-DUP; χ2 = 0.84; p = 0.36) or age (36 years old in the short-DUP vs 37 years old in the long-DUP; p = 0.52) between groups.

A greater number of people in the long-DUP group were unemployed (χ2 = 18,136, p = 0.02) compared to the short-DUP group. A&E visits were significantly higher in short-DUP group (71.8% vs 28.2%, χ2 = 8.82; p = 0.003). No significant differences were found between groups in terms of hospital admission or duration of stay.

The rate of responders using the PANSS remission criteria proposed by Andreasen was 82.7% at 3 months. Non-responders were 15.9% in the short-DUP vs 20.8% in the long-DUP (p = 0.43). Non-significant differences were found.

At 3 months, the rate of patients who scored more than 70 points on the EEAG scale was 71.5%. Non-significant differences were found (70% short-DUP vs 74% long-DUP; p = 0.49) between both groups.

**Conclusions:**

We observed that the percentage of non-responders at 3 months is higher in the group with a larger DUP. At 3 months, patients within the early intervention program showed a high level of functioning regardless of the duration of untreated psychosis.

**Disclosure of Interest:**

None Declared

